# Surveillance in a Neonatal Intensive Care Unit Allowed the Isolation of a Strain of VIM-Producing *Pantoea brenneri*

**DOI:** 10.3390/antibiotics12010098

**Published:** 2023-01-06

**Authors:** Cristina Merla, Irene Mileto, Stefano Gaiarsa, Cristian Achille, Stefano Ghirardello, Marta Corbella, Fausto Baldanti, Patrizia Cambieri

**Affiliations:** 1 UOC Microbiologia e Virologia, Fondazione IRCCS Policlinico San Matteo, 27100 Pavia, Italy; 2 Scuola di Specializzazione in Microbiologia e Virologia, Università degli Studi di Pavia, 27100 Pavia, Italy; 3 Neonatologia e Terapia Intensiva Neonatale, Fondazione IRCCS Policlinico San Matteo, 27100 Pavia, Italy; 4 Department of Clinical: Surgical, Diagnostic and Pediatric Sciences, Università degli Studi di Pavia, 27100 Pavia, Italy

**Keywords:** verona integron β-lactamase, screening for carbapenemase-resistant Enterobacterales, newborn, neonatal intensive care unit, *Pantoea brenneri*, VIM

## Abstract

Here, we describe the isolation of a strain of the genus *Pantoea* encoding a VIM carbapenemase, the first to our knowledge. The strain, isolated from a rectal swab of a 10-day-old newborn admitted to a neonatal intensive care unit (NICU), was identified through whole-genome sequencing analyses as *Pantoea brenneri*. The strain harbored the carbapenemases gene *bla_VIM-1_*. The prompt application of contact measures and the isolation of the newborn prevented the dissemination of VIM-producing *P. brenneri* and of the plasmid carrying the VIM-1 gene to other newborns.

## 1. Introduction

*Pantoea* spp. is a well-known plant pathogen [1] belonging to the Enterobacteriaceae family. A taxonomical revision in the *Pantoea agglomerans* complex amended the description of the genus *Pantoea* and described *Pantoea septica* sp. nov., *Pantoea eucrina* sp. nov., *Pantoea brenneri* sp. nov., *Pantoea conspicua* sp. nov., and *Pantoea cypripedii* comb. nov. [2]. *Pantoea* spp. rarely causes opportunistic infections [3], occurring mainly due to the contamination of wounds, or through hospital-acquired infection in individuals with a weak immune system such as newborns [4,5]. In preterm newborns, contamination of the parenteral nutrition with *Pantoea* spp. has been reported as a cause of bacteremia [6].

Carbapenem-resistant Enterobacteriaceae are increasingly common worldwide with growing numbers of people being infected by these organisms associated with significant morbidity and mortality. Knowledge of carbapenemase type is crucial for antibiotic treatment because not all β-lactamase inhibitor combinations are active against all carbapenemase types. Indeed, metallo-β-lactamases such as Verona integron metallo-β-lactamase (VIM) are not inhibited by beta-lactamase inhibitors such as clavulanic acid, tazobactam, avibactam, and vaborbactam, leaving few options for therapy. VIM-producing Enterobacterales are reported globally, and in Europe especially in the Mediterranean area [7,8,9]. Nevertheless, no *Pantoea* spp. bearing carbapenemase genes have been reported until now, while extended-spectrum beta-lactamase (ESBL)-producing strains bearing *bla_CTX-M_* and *bla_TEM-1_* genes have been reported [10,11,12].

In this case report, we report the first isolation of *Pantoea brenneri* carbapenem-resistant bearing VIM carbapenemase. The strain was isolated from a newborn who was colonized. 

## 2. Case Description

A pre-term male baby (34 weeks) born on 1 March 2022 by cesarean section from a diabetic mother was admitted to the Neonatal Intensive Care Unit (NICU) of tertiary hospital Fondazione IRCCS Policlinico San Matteo in Pavia (Italy) due to respiratory distress. The newborn underwent oxygen therapy with nasal continuous positive airway pressure (nCPAP) because a congenital pneumonia was suspected. Ampicillin and gentamycin were started as empirical treatment at admission as per internal procedures. Ceftazidime was also started and blood cultures collected due to the critical clinical condition of the patient and the suspicion of early-onset sepsis [13]. Total parenteral nutrition was administered by the central venous route until day 3, when enteral feeding through an orogastric tube was started. Ceftazidime was stopped after 72 h according to the American Academy of Pediatrics [14] while the administration of ampicillin and gentamycin was continued. Surveillance rectal swabs for the screening of ESBL-producing Enterobacterales collected on days 3 and 7 were negative.

On day 10, *Pantoea* spp. (6775PV) was cultured from the surveillance rectal swab. The patient was spatially isolated and contact isolation precautions were taken including dedicated materials such as gauzes, band-aids, syringes, and disinfectants, dedicated nursing personnel 24/7, and a more accurate environmental cleaning performed more frequently with sodium hypochlorite 1000 ppm with particular attention for surfaces that could be touched by multiple people such as door handles and electromedical equipment. Disposable materials were used when possible, and disinfecting bottles or wipes were placed near ready-to-use equipment such as the imaging ultrasound system. Hand hygiene practices were implemented for all of the personnel of the NICU, and ESBL surveillance for all of the other newborns of the NICU continued with rectal swabs performed at admission and weekly thereafter, as defined in the hospital operating procedures for multidrug resistance (MDR) bacteria surveillance. On day 12, ampicillin and gentamycin were stopped as well. *Pantoea* spp. was also grown from the rectal swab collected on day 14. On day 19, the newborn male was discharged in generally good health with normal cardio-respiratory parameters.

## 3. Microbiological Investigations

**Figure 1:**
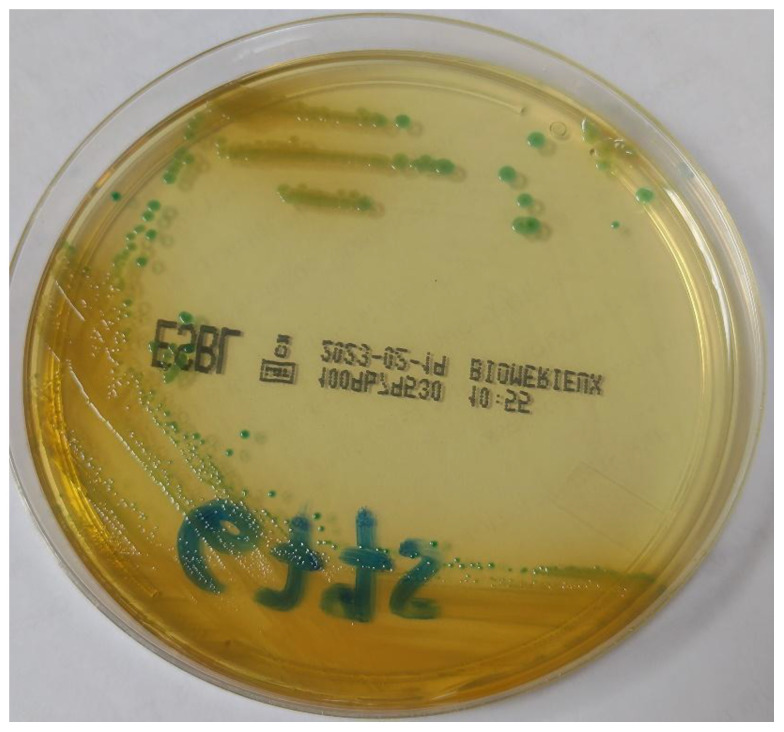
Morphology of the colonies of *Pantoea brenneri* on chromID ESBL agar (BioMerieux, Marcy-l’Etoile, France).

Surveillance rectal swabs were cultured on chromID ESBL Agar (BioMerieux, Marcy-l’Etoile, France). Flat blue-green colonies grew after incubation at 37 °C for 18 h (Figure 1). Species identification was performed through MALDI-TOF mass spectrometry (Bruker Daltonics GmbH, Bremen, Germany) equipped with Bruker Biotyper 3.1 database. The isolate 6775PV was identified as *Pantoea agglomerans* (score 1.828), but scores between 1.7 and 1.99 indicate “probable genus identification”, thus the strain was reported as *Pantoea* spp.

The susceptibility profile determined using the NMIC-402 panel of the Phoenix M50 BD automated system (Becton Dickinson, Franklin Lakes, NJ, USA) and Sensititre DKMGN (ThermoFisher Scientific, Rodano, Italy) showed resistance to cephalosporins and carbapenems (Table 1). Fosfomycin was tested via fosfomycin agar dilution (Liofilchem, Roseto degli Abruzzi, Italy). The presence of VIM carbapenemase was assessed using NG test CARBA 5 (NG Biotech, Guipry, France) and confirmed with the Cepheid Xpert^®^ Carba-R assay (Cepheid, Sunnyvale, CA, USA).

Genomic DNA of the isolate was extracted with a blood and tissue kit (Qiagen, Düsseldorf, Germany) following the manufacturer’s instructions for short-read sequencing. Short reads were obtained by an Illumina MiSeq platform with a 2 × 150 paired-end run, after a Nextera XT library preparation step (Illumina Inc., San Diego, CA, USA). 

**Figure 2:**
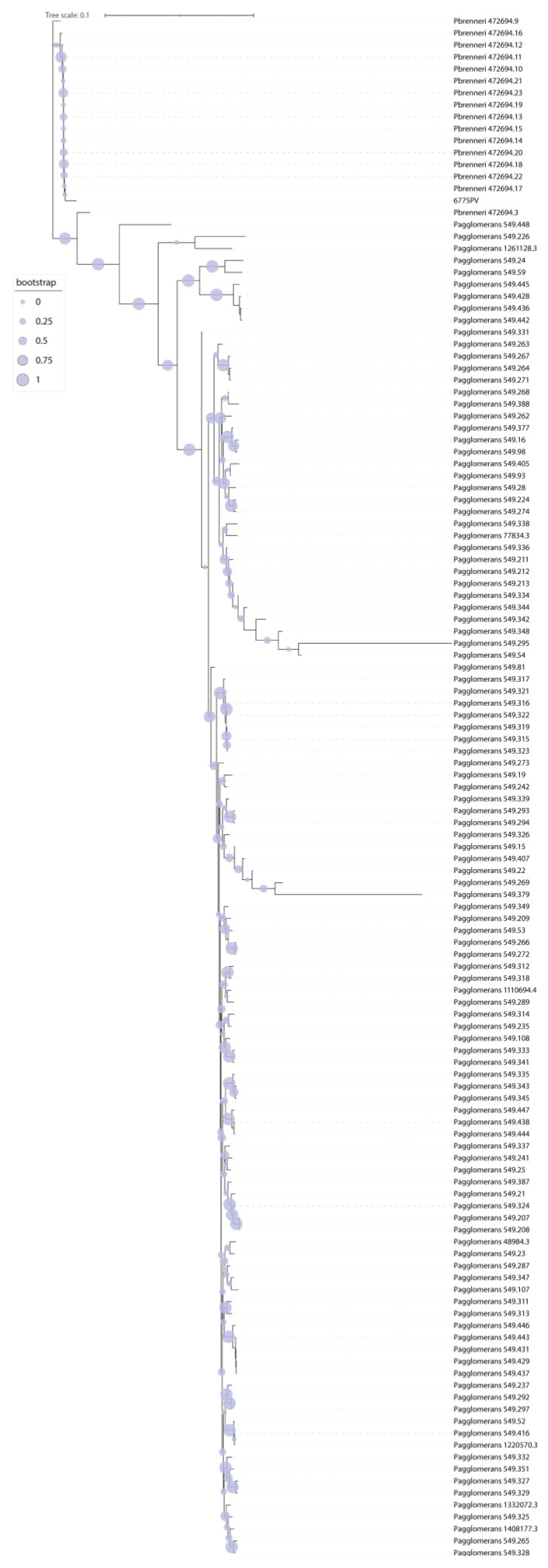
Phylogeny of the genome of isolate 6775PV and all high-quality genomes of the *P. agglomerans* and *P. brenneri* species. The Phylogeny was obtained using the software JolyTree. Bootstrap values are represented with colored dots of variable size on the tree branches.

Genomes were assembled using Shovill 1.1. The genome of strain 6775PV is 4,756,675 bp in length with Ncontig (>200 bp) = 79 and N50 = 148,391. The software PGAP identified the genome as *Pantoea brenneri*, a species that is not present in the Bruker Biotyper 3.1 database. Thus, a phylogeny was performed using the software Jolytree on the genome of our isolate and all high-quality genomes of both *P. agglomerans* and *P. brenneri*, retrieved from the PATRIC database (https://www.patricbrc.org/, accessed on 22 June 2022). The resulting tree (Figure 2) indicates strain 6775PV clusters with the genomes of *Pantoea brenneri.* The closest genome to 6775PV in the phylogeny was isolated from a surface in the International Space Station in 2015 [15].

Resistance genes were searched using ResFinder 4.1 [16] with a coverage threshold of 90% and identity threshold of 100%, and plasmids were searched with PlasmidFinder 2.1 [17] with a coverage threshold of 100% and an identity threshold of 95%. The genome 6775PV harbored the resistance genes *bla_VIM_-_1_*, *aa’(6’)-Il,* and *qnrS1* encoding, respectively, for resistance to carbapenems, aminoglycosides and fluoroquinolones, and IncN2 plasmid replicon type. 

## 4. Discussion

Here, we describe the first isolation of a strain of the genus *Pantoea* bearing VIM-1 carbapenemase isolated from a rectal swab of a 10-day-old newborn The isolation of a strain of *Pantoea* spp. resistant to carbapenems, one of the last options to treat multidrug-resistant Gram-negative organisms, is particularly worrisome. Indeed, plasmids carrying *bla_VIM_* usually also carry other genes conferring resistance to antibiotics, including aminoglycosides, macrolides, and sulfamethoxazole, making these strains MDR, further limiting treatment options.

The colonization by *Pantoea* spp. as well as by other Enterobacterales carrying VIM carbapenemases, even if asymptomatic, can be considered a risk factor to develop infection. Hence, the role of surveillance in infection control programs is crucial since contact isolation measures reduce the chances of patient-to-patient transmissions, and patient outcomes improve due to the earlier availability of the susceptibility profile of the pathogens, resulting in prompt administration of appropriate antimicrobial therapy. 

Bacteria belonging to the genus *Pantoea* spp. are considered the cause of opportunistic human infections, mostly wound infections but also bacteremia. Indeed, the only report of infections caused by wild-type *P. brenneri* was a nationwide sepsis outbreak in the USA in 1971 [2]. Bacteremia caused by Pantoea spp. has been described in adults in Japan [18] and the USA [19]. In newborns, bacteremia caused by wild-type *Pantoea* spp. has been previously described in Brazil and India [20,21]. Sepsis and wound infections caused by carbapenem-resistant *P. agglomerans* in newborns were reported in Turkey and in Yemen [10,22], but no indication of the class of resistance mechanism is given in either study.

The only case of Pantoea infection reported in Europe previously regarding ESBL-producing *Pantoea agglomerans* was described from clinical specimens of pediatric patients in a Bulgarian hospital [8], and recently, in the same NICU as this study, where ESBL-producing *Pantoea calida* colistin-susceptible but bearing the *mcr-9* gene for colistin resistance was isolated from a newborn [23]. In Italy, wild-type strains of *Pantoea* spp. were reported from blood cultures of patients admitted to oncology and ICU [24,25] and from a contaminated port-a-cath [26]; but in both studies, the isolates were wild type.

Although *Pantoea* spp. is not considered a pathogen, horizontal transfer of resistance genes mediated by plasmids and other mobile elements occurs especially in healthcare settings where antimicrobial use increases selection pressure on bacterial populations. In this study, strain 6775PV harbored a plasmid that belongs to the IncN incompatibility group, one of the most common mobile genetic platforms for the spreading of resistance genes among Enterobacteriaceae. A multispecies cluster of Enterobacterales carrying VIM-1 carbapenemase was isolated in 2019 from rectal swabs and blood cultures of patients of a teaching hospital in Rome (Italy) [27]. However, this cluster is characterized by IncA plasmid.

The emergence and dissemination of carbapenem-resistance mechanisms represent a global public health concern, resulting in infections associated with poor outcomes, high mortality rates, and limited treatment options. The emergence of metallo-β-lactamase-producing isolates requires prompt surveillance especially of colonization by carbapenem-resistant isolates belonging also to species not usually considered pathogenic, which can act as reservoirs and can promote inter- and intra-care setting dissemination.

In this case, the strict surveillance and the prompt application of infection control measures have prevented the spreading of VIM-1-producing *P. brenneri* and the dissemination among other bacterial species of the *bla_VIM-1_* carbapenemase gene. Infections caused by VIM-producing Enterobacterales are worrying especially for critical patients like newborns in NICU. In the future, epidemiologic studies should be performed to investigate the diffusion of carbapenem resistance among species not usually considered in surveillance, especially in critical patients, and bundled infection control measures, education, and training should be implemented to limit and control the spreading of carbapenem resistance.

## Figures and Tables

**Table 1:** Antibiotic susceptibility profiles of the 6775PV isolate. Interpretation was performed according to EUCAST breakpoints (version 12, 2022) for Enterobacterales: S was used for susceptible, I for susceptible increased exposure and R for resistant. Breakpoints are displayed under each antibiotic name.

Strain	AMC S ≤ 8 R > 8	AMP S ≤ 8 R > 8	P/T S ≤ 8 R > 8	FEP S ≤ 1 R > 4	CTX S ≤ 1 R > 1	TAZ S ≤ 1 R > 4	CAZ S ≤ 8 R > 8	C/T S ≤ 2 R > 2	CIP S ≤ 0.25 R > 0.5	LVX S ≤ 0.5 R > 1	ERT S ≤ 0.5 R > 0.5	IM S ≤ 2 R > 4	MEM S ≤ 2 R > 8	ATM S ≤ 1 R > 4	FOS S ≤ 32 R > 32	COL S ≤ 2 R > 2	AK S ≤ 8 R > 8	CN S ≤ 2 R > 2	SXT S ≤ 2 R > 4
6775PV	>64/2 (I)	>8 (R)	>32/4 (R)	>8 (R)	>8 (R)	>16 (R)	<16/4 (R)	>32/4 (R)	0.5 (I)	≤0.5 (S)	1 (R)	16 (R)	4 (R)	≤0.5(S)	>64 (R)	≤0.25 (S)	≤4 (S)	≤1 (S)	≤1/19 (S)

AMC: amoxicillin-clavulanate, AMP: ampicillin, P/T: piperaciilin/tazobactam, FEP: cefepime, CTX: cefotaxime, TAZ: ceftazidime, CZA: ceftazidime-avibactam, C/T: ceftolozane-tazobactam, CIP: ciprofloxacin, LVX: levofloxacin, ETP: ertapenem, IM: imipenem, MEM: meropenem, ATM: aztreonam, FOS: fosfomycin, COL: colistin, AK: amikacin, CN: gentamycin, SXT: trimethoprim/sulfamethoxazole.

## Data Availability

Genome assembly data are available at NCBI under BioProject ID PRJNA842925 (BioSample accession: SAMN28693404).
